# Assessment of caregivers’ perspectives regarding speech-language services in Saudi Arabia during COVID-19

**DOI:** 10.1371/journal.pone.0253441

**Published:** 2021-06-22

**Authors:** Nisreen Naser Al Awaji, Alanoud A. Almudaiheem, Eman M. Mortada

**Affiliations:** 1 Department of Health Communication Sciences, College of Health and Rehabilitation Sciences, Princess Nourah Bint Abdulrahman University, Riyadh, Saudi Arabia; 2 King Abdullah Specialized Children Hospital-King Abdulaziz Medical City, Riyadh, Saudi Arabia; 3 Health Sciences Department, College of Health and Rehabilitation Sciences, Princess Nourah Bint Abdulrahman University, Riyadh, Saudi Arabia; 4 Community, Environmental & Occupational Medicine Department, Faculty of Medicine, Zagazig University, Zagazig, Egypt; University of Oklahoma Health Sciences Center, UNITED STATES

## Abstract

**Background:**

As a consequence of stay-at-home and other lockdown measures, such as social distancing, all health care service provisions during the COVID-19 pandemic have been affected, including the provision of speech therapy. Telehealth services can play a major role in maintaining access to health care, help speech and language pathologists (SLPs) overcome physical barriers by providing patients and caregivers with access to health care, and limit the discontinuity of patient care. To have a better understanding of the changes that have occurred in these services during COVID-19, this research was conducted to explore the nature and current situation of speech-language services in Saudi Arabia based on caregivers’ perspectives. It also investigated whether changes have occurred in these services during the COVID-19 lockdown. The study also determined the perception of caregivers in delivering SLS sessions remotely.

**Method:**

A cross-sectional study was conducted with 385 caregivers in Saudi Arabia. An online survey asked whether children were experiencing any SLS problems and if they had received any intervention. The survey also assessed the perception of changes in service during the COVID-19 lockdown and the perceptions, acceptance, and willingness of the caregivers to deliver telehealth speech services in Saudi Arabia.

**Results:**

About 50% of the respondents had or were suspected to have a child with SLS problems, and just over half of them had accessed SLS services. Most of the respondents reported suspension of therapy sessions as a response to the COVID-19 pandemic. While the respondents had little experience using telehealth prior to the pandemic, they generally showed a willingness to use telehealth in therapy sessions, expressing a preference for video calls over other options.

**Conclusion:**

The study revealed that SLS services in Saudi Arabia are limited and that accessing these services is challenging. Alternative service delivery using remote services could help caregivers overcome such challenges. When telehealth was introduced as an option for service delivery, the caregivers showed welcoming responses, particularly with video calls.

## Introduction

Epidemiological data on different disabilities in Saudi Arabia reported that 667,280 out of 20,064,970 Saudis have disabilities, with speech and communication disorders being the second most common type [1]. An earlier survey conducted by Milaat et al [2] in the eastern region found that 3.6% of children have functional disabilities, with communication disorders being the most common.

Different studies have reported the various impacts of speech and communication disorders, including social difficulties, and problems with reading, spelling, and mathematics [3, 4]. Late intervention may lead to long-term impacts for children with speech and language problems, which may persist for at least 28 years [5]. Thus, the UK and US governments have acknowledged the negative consequences of speech and communication disorders and have recognized the necessity of accessing Speech-Language and Swallowing (SLS) services for children with these disorders [6, 7]. Although the Saudi government has made improvements in relation to rehabilitation services, the development of SLS services has not received a high priority, probably due to a lack of awareness of the services provided by speech-language pathologists (SLPs) [8].

While there is clearly a shortage of speech-language services in Saudi Arabia, only a limited number of studies have documented this. Alquraini [9] reported that speech-language services were only available in health care settings and in the private sector, whereas educational and social service institutions lack SLS services. This has led to a shortage of staff and uneven distribution of resources and facilities [8]. The imbalance in the geographical distribution of speech-language pathologists (SLPs) across the country is also a significant issue. This has been anticipated, as most health care providers prefer to live in urban cities, where they have both professional and social advantages [10–12]. On the other hand, in a single study that reported parents’ perspectives on SLS services in Saudi Arabia, parents reported a lack of rehabilitation services for their children: only one-third of them had the service [2].

Evidence indicates that children with SLS disorders usually need at least one session per week and that sessions could last for several weeks with a qualified clinician [13–15]. However, despite the regular and frequent needs of services among these children, not all families can access the service. The reasons for this include the existence of waiting lists, unfamiliarity with the ways in which the services are accessed, and a lack of awareness of the benefits of these services [16]. Furthermore, families who live in rural areas may experience different challenges when accessing these services, which may be attributed to long distances and their associated difficulties, including the cost of transportation and scheduling conflicts.

A potential solution to overcoming the barriers of accessing SLS services caused by a shortage of SLPs, geographical distance, or impaired mobility is to use telehealth as a service model for SLPs to deliver their services [17] [ASHA]. ASHA has defined telehealth as “the use of telecommunications technology to deliver professional services at a distance by linking clinician to client or clinician to clinician for assessment, intervention, and/or consultation” [18].

The literature provides evidence that telehealth is an effective and feasible mode of service delivery that can be used with a wide range of patients [19]. There is an extensive literature on the notion of the use of telehealth in the assessment, diagnosis, and treatment of different speech and communication disorders, especially for those who have difficulty accessing these services [19–21].

Furthermore, the recent dilemma faced by health care systems worldwide with the emergence of the COVID-19 pandemic and so-called social distancing have forced health care providers to convert their services to telehealth [22, 23]. In Saudi Arabia, it appears from the literature that adoption of telehealth was slow before the COVID-19 pandemic [24, 25]; however, various health care settings have accelerated the use of telehealth services during the pandemic so that patients can easily access these services from their homes [26]. However, the utility of telehealth should not be limited to the COVID-19 pandemic; it needs to be considered as a service option and a possible solution in removing barriers and creating opportunities, especially for patients and families who have difficulty accessing the service.

Families’ perceptions regarding the use of telehealth services have been investigated in different studies (i.e. [27, 28]; those who used telehealth reported that it was accessible and feasible. Other studies have identified a number of disadvantages and barriers to the use of telehealth, including difficulty in establishing rapport between clinicians and children or families, internet and technical issues, and inability to use some materials in therapy [29–31]. These barriers could limit the adoption of telehealth among stakeholders, including patients and caregivers [22]. In addition to the importance of investigating the perceptions of caregivers who have used telehealth, exploring the perceptions of those who have not used the service may also help to increase interest and awareness in telehealth services and to identify difficulties that may be experienced by caregivers or families when using telehealth [32].

### Objectives of the study

- To explore, based on caregivers’ perspectives, the nature and current situation of speech-language services in Saudi Arabia;
- To assess if changes occurred in SLS services during the COVID-19 lockdown;
- To determine the effectiveness, applicability, and usefulness of providing speech and swallowing sessions remotely.

## Materials and methods

### Participant and procedure

A cross-sectional study was conducted involving 385 caregivers of children aged from 0–14 years. Responses were collected in the period between 24 June 2020 and 19 July 2020, mainly at the end stages of the lockdown in Saudi Arabia. The survey was generated with the Google survey tool (Google Forms) and distributed by all members of the current research team to different social media channels (Twitter, Facebook, WhatsApp, Telegram) and different societies, including the Saudi Society of Speech-language Pathology and Audiology (SSSPA) and Saudi Craniofacial Anomalies to advertise the survey only. The inclusion criteria were: agreement to participate in the study, current residence inside Saudi Arabia, and being a family member of a child aged 0–14 years.

The required sample size of this study was calculated to be 384 responses (supplement 1). Four hundred and seven caregivers responded to the online questionnaire. After excluding 22 ineligible responses and those with incomplete data, the final sample consisted of 385 participants. Their personal characteristics are summarized in Table 1. Among all respondents, 80% were mothers (n = 300), 6% were fathers (n = 24), and 70.9% % had attained a bachelor’s degree or higher educational level. A summary of the personal characteristics of the respondent caregivers is presented in Table 1.

**Table 1:** Personal characteristics of respondent caregivers.

Characteristics	No	%
**Relationship with the child(n = 385):**		
○Mother	300	77.9
○Father	24	6.3
○Others	61	15.8
**Educational level of the caregiver(n = 385):**		
○Elementary school	41	10.7
○Intermediate school	3	0.7
○High school	68	17.7
○Bachelor degree	189	49.1
○Higher education	84	21.8
**Place of residence**		
○ Riyadh	250	65.1
○ Jeddah	51	13.3
○ Makkah	12	3.1
○ Taif	2	.5
○ Dammam	10	2.6
○ Khobar	14	3.6
○ Alhassa	14	3.6
○ Najran	7	1.9
○ Others	25	6.5

### Survey design

**Fig 1:**
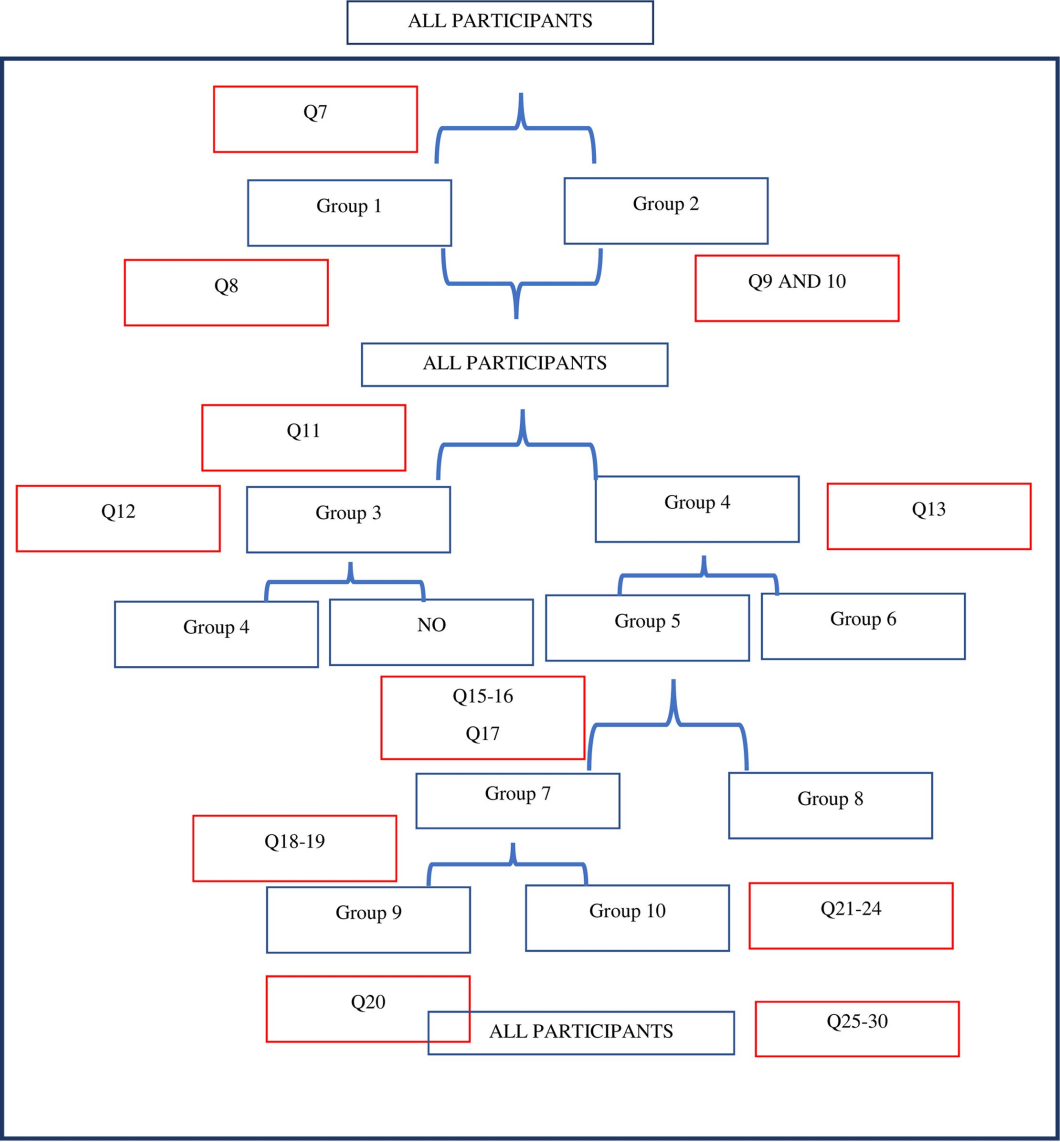
Flow chart illustrating the skipping pattern in the used survey.

The self-report survey was developed by the research team to indicate whether the children had any SLS problems, and if they had received any intervention. The survey also assessed the perception of changes in the service during the COVID-19 lockdown and the perceptions, acceptance, and willingness of the caregivers to deliver telehealth speech services in Saudi Arabia. The survey consisted of three main sections:

1. In the first section of the survey, general information regarding the child and caregiver were obtained: caregiver’s relationship to the child (Q1), city of residence (Q2), level of education (Q3) and child’s age (Q4), health condition (Q5), and any history of hearing problems (Q6).
2. The second section included a question about the presence of any speech and language problems (Q7). Depending on their answers, the participants were then divided into one of two groups: those who answered “yes” were assigned to Group 1, while those who answered “no” or “not sure” were assigned to Group 2 as illustrated in Fig 1.

Group 1 were then asked to choose from a list of speech and language problems (Q8): shows difficulty understanding requests, has limited receptive vocabulary, misarticulates speech sounds, stutters, has hyper nasality, has a voice problem, or others from which he must specify. Group 2 were asked another set of questions: “Have you ever been told that your child has reading and/or spelling problems?” (Q9) and “Have you ever been told that your child has poor speech intelligibility?” (Q10).

Both groups were then asked if they had a speech therapy clinic in their city of residence (Q11). They were divided into one of two groups, depending on their answers. “Those who answered “yes” were assigned to Group 3 and asked, “Do you know where the nearest speech therapy clinic is located?” (Q12). Those who answered “no” were assigned to Group 4 and asked, “Did your child ever visit the speech therapy clinic?” (Q13). Depending on their answers, they were assigned to one of the two subdivisions of Group 4: Those who answered “yes” were assigned to Group 5, while those who answered “no” were assigned to Group 6.”

Group 5 (“yes, my child visited a speech therapy clinic”) were asked a set of questions to determine: the city where the speech therapy clinic they visited was located (Q14), the frequency of speech therapy sessions (Q15), how long the child received speech therapy services (Q16), and if the speech and language sessions had been stopped (Q17).

Group 5 was divided into 2 groups depending on their answers to Q17; those who answered “yes” as to whether the service had stopped were assigned to Group 7, while those who replied that the speech service continued were assigned to Group 8.

Group 7 were asked, “When was his last speech therapy session?” (Q18) and “Why were the speech therapy sessions stopped?” (Q19). Depending on their answers to Q19, the members of Group 5 were subdivided into one of 2 groups. Those who answered, “Difficulties in having this service due to COVID-19” were assigned to Group 9, while those who answered, “My child has overcome his speech problem, my child did not show any improvement from the speech therapy sessions, there are no speech therapy clinics around” were assigned to Group 10.

Group 9 were asked to specify the difficulties of having the service during COVID-19 (Q20): fear of getting infected, the center/sessions are temporarily suspended, financial problems due to COVID-19, applying quarantine, lockdown times, low immunity of the child.

If their answer was “No, the speech service continued” (Group 8), they were asked to provide information about the type of speech service provided (Q21), regular speech therapy sessions in the clinic, to provide the recorded speech therapy sessions sent from the speech therapist, the home therapy sessions provided by the therapist, virtual video sessions, phone call guidance and counseling, a therapy program to be applied at home with full support from the speech therapist when needed, or a therapy program to be applied at home with minimal support from the speech therapist, when needed.

The members of Group 8 were also asked their opinion about the services provided: “Did you benefit from the service?” (Q22) and “Did you face any difficulties applying the instructions provided?” (Q23). The participants who faced difficulties applying the instructions were asked to list the difficulties (Q24).

All participants were asked a set of questions to elicit their general opinions: “Can speech therapy sessions be provided through phone calls (Q25) and video calls (Q28)?”, “Can phone calls (Q26) and video call (Q29) sessions replace regular sessions in the clinic?”, and “Is the information provided through phone calls (Q27) and video calls (Q30) beneficial?”

3 In the last section, all participants were asked their opinions about having speech therapy sessions through Tele-practice (Q31).
  Experts assessed the items of the questionnaire regarding the necessity, through giving score for each question (1–3) for " nit necessary, somewhat necessary and necessary" in respective order.
  Also, the relevance of the questions was assessed by the same experts by giving scores from (1–4) for" not relevant, somewhat relevant, more relevant, highly relevant". Accordingly, the Content Validity Ratio (CVR) was calculated "0.81" and Content Validity Index (CVI) was also calculated "0.84". These levels are considered acceptable.

### Ethical considerations

The study was approved by the Institutional Review Board (IRB) at Princess Nourah Bint Abdulrahman University (IRB log number: .020–0251), Riyadh, Saudi Arabia. Formal letter was attained from the university. Respondents were provided a description of the study and were assured of the confidentiality of their information. A consent statement was also provided at the beginning of the survey to guarantee the respondents’ informed consent and agreement to contribute to the study.

### Data analysis

Collected data were analyzed using SPSS (V. 20). Descriptive Statistics were represented as frequencies and percentages for categorical variables. Data were presented graphically with Microsoft Excel using the data obtained. A chi-square test was used to measure the association between having disordered children and the desire of caregivers for telehealth sessions. The results were considered statistically significant at p ≤ 0.05.

## Results

**Fig 2:**
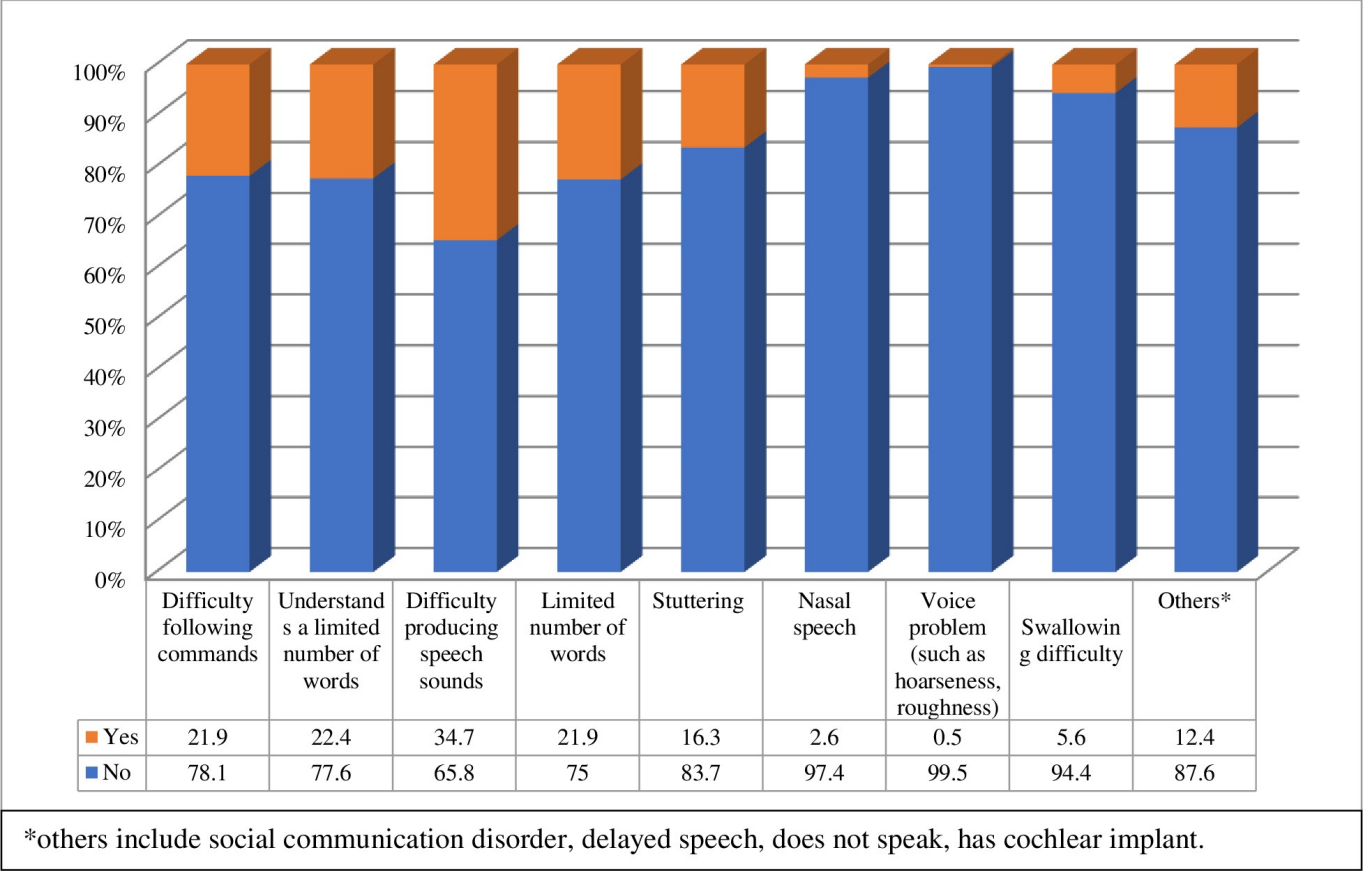
Kinds of speech, language, or swallowing disorders of the children (n = 196).

Caregivers were asked whether they had children with speech, language, or swallowing disorders (SLS) (Q1); 45% of the respondents said “Yes”, 49% said “No”, and only 6% said “Maybe” (Table 2). The age of children who had or might have had problems in SLS ranged from under 2 to 14 years old. Most were aged 3–5 years (n = 68/196, 34.7%) and 6–8 years (n = 56/196, 28.6%). Sixteen percent of these children had hearing problems (Q3), while other health problems were reported in 19% of the children (Q2). The types of SLS problems reported by respondents included difficulty producing speech sounds (34%), difficulty following commands (23%), produce a limited number of words (23%), and being able to understand only a limited number of words (22%) (Fig 2).

**Table 2:** Characteristics of children.

	Characteristics		No		%
	**Q1. Do you have any child with speech, language or swallowing disorders? (n = 385)**				
	○ No	189		49.1	
	○ Yes	173		44.9	
	○ May be	23		6.0	
‘Yes’ to Q1	**How old is your child (years) (n = 196):**				
	○ 0–2	12		6.1	
	○ 3–5	68		34.7	
	○ 6–8	56		28.6	
	○ 9–11	38		19.4	
	○ 12–14	22		11.2	
	**Q2. Does your child have any other health problems? (n = 196)**				
	○ No	158		80.6	
	○ Yes	38		19.4	
	**Q3. Does your child have hearing problems? (n = 196)**				
	○ No	165		84.2	
	○ Yes	31		15.8	
‘No’ to Q1	**Q4. Has anyone ever told you that the child may have reading or spelling, pronouncing and swallowing problems? (n = 189)**				
	○ No	162		85.7	
	○ Yes	27		14.3	
	**Type of the problem(n = 27)**				
	○ Reading or spelling problems	12		44.4	
	○ pronouncing sounds	14		51.6	
	○ Swallowing problem	1		3.7	

Respondents who answered “No” to Q1 were asked, “Has anyone ever told you that the child may have reading or spelling, pronouncing, and swallowing problems?” (Q4). Fourteen-point three percent (n = 27/189) replied “Yes”. Of these, 44.4% (n = 12/27) reported problems in reading and spelling, 51.6% (n = 14/27) reported problems in pronouncing sounds, with only one child (3.7%, n = 1/27) presenting with swallowing problems.

### SLS services

Table 3 presents information regarding the need and availability of SLS services. A question was asked of the 196 respondents who had or thought they had a child with an SLS disorder: “Are there speech/swallowing clinics available in your area/city?” (Q5). Only 12.8% answered “Yes”, 54.1% said “No”, and the rest answered, “Do not know”. Then, another question was asked: “Has your child visited speech/swallowing clinics before?” (Q6); just over half responded “Yes” (n = 110/196, 56.1%), and 48.1% (n = 52/110) of these said that they had a therapy session once per week (Q7). When the participants were asked, “How long is/was your child in speech/swallowing therapy?” (Q8), 32.7% (n = 36/110) had therapy sessions lasting for less than two months, 25.5% (n = 28/110) had sessions for a period between two and six months, 10% (n = 11) had sessions from 6–12 months, and around 23% (n = 25/110) had been attending sessions for more than a year.

**Table 3:** Need and availability of SLS therapy sessions before COVID-19 pandemic.

Characteristics	No	%
**Q 5. Are there speech/swallowing clinic available in your area/ city? (n = 196)**		
○ Yes	25	12.8
○ No	106	54.1
○ Don’t know	65	33.2
**Q 6. Child has ever visited speech /swallowing clinics before (n = 196)**		
○ No	86	43.9
○ Yes	110	56.1
**Q 7. Frequency of getting speech / swallowing sessions (n = 110)**		
○ Once a week	52	47.3
○ Once every two weeks	11	10.0
○ Once every 3 weeks	10	9.1
○ once a month	14	12.7
○ Others ^a^	23	20.9
**Q 8. How long is/was your child in speech/swallowing therapy before discharge? (n = 110)**		
○ Less than two months	36	32.7
○ Between 2 and 6 months	28	25.5
○ From 6 months to 12 months	11	10.0
○ More than 12 months	25	22.7
○ Others^b^	10	9.1

^a: twice/week, once, none, uncertain, once/6months.^

^b: once, never, 3 years.^

### Changes in SLS services during COVID-19 pandemic

**Fig 3:**
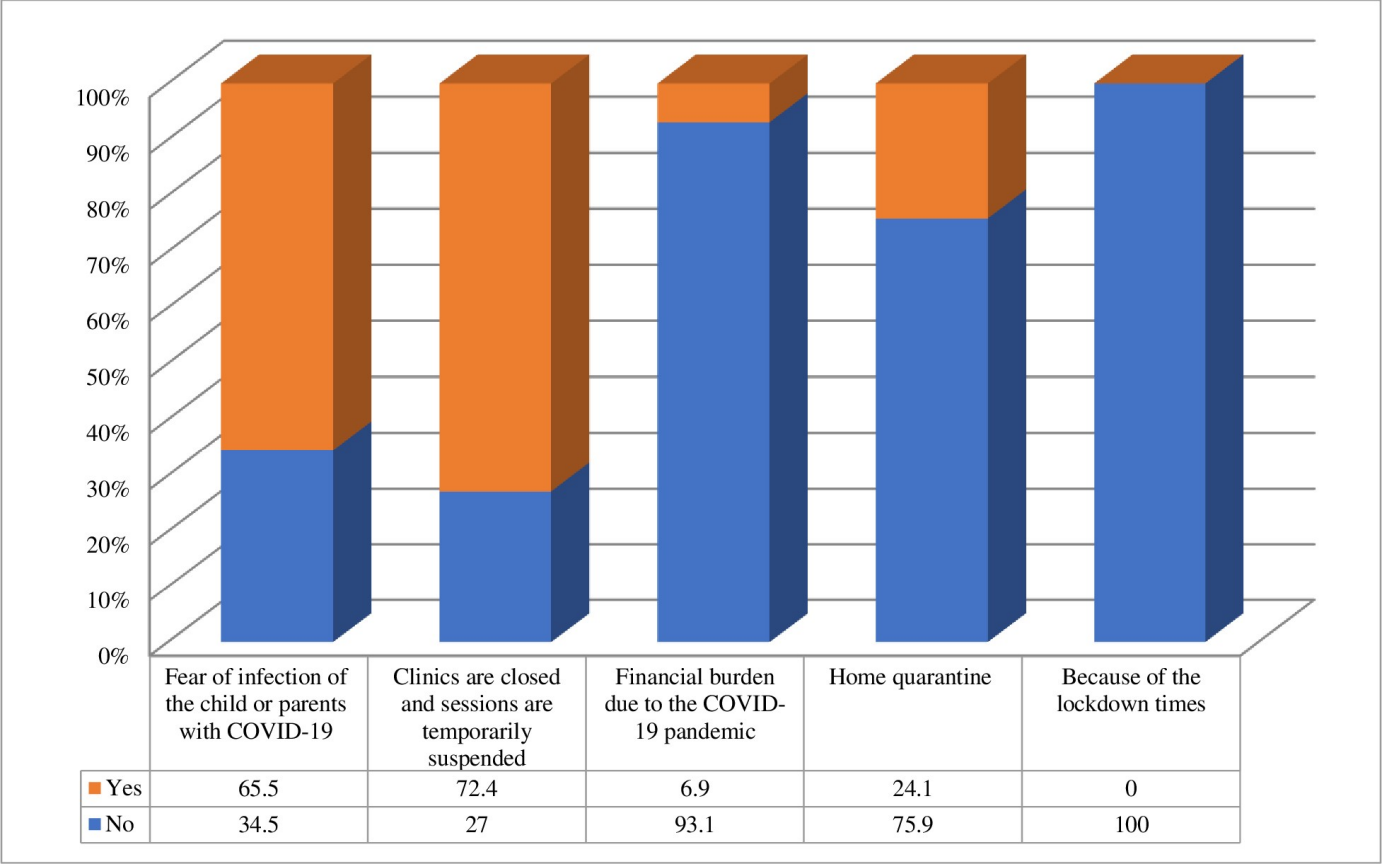
Reasons for difficulty accessing services due to COVID-19 pandemic (n = 35).

Table 4 shows the responses to questions assessing changes in speech and swallowing services during the COVID-19 pandemic. Asked if therapy sessions had stopped as a response to COVID-19 (Q9), most respondents said “Yes” (80.9%, n = 89/110), and the reported reasons were: difficulty accessing services due to COVID-19 pandemic crisis (39.3%), speech problem was resolved (22.5%), did not find any improvement in the child’s condition (14.6%), and no available speech clinics (14.6%). Those who had difficulty accessing services due to the pandemic were asked about the reason/s for such difficulty, to which they replied: clinics are closed and sessions are temporarily suspended (72.4%), fear of infection of the child or parents with COVID-19 (65.5%), home quarantine (24.1%), and, lastly, financial burden due to the pandemic (6.9%) (Fig 3).

**Table 4:** Changes in speech and swallowing services during the COVID-19 pandemic.

Characteristics	No	%
**Q 9. Have speech, language or swallow therapy sessions stopped? (n = 110)**		
○ No	21	19.1
○ Yes	89	80.9
**Q 11. Why did speech / swallowing therapy sessions stop? (n = 89)**		
○ Difficulty accessing services due to COVID-19 pandemic crisis.	35	39.3
○ Speech problem was solved.	20	22.5
○ I did not find any improvement in my child’s condition.	13	14.6
○ There are no available speech clinics	13	14.6
○ Others	8	9.0
**Q 12. What are the benefits from the service provided by the therapist during the COVID-19 pandemic (n = 21)**		
○ No	5	23.8
○ Yes	16	76.2
**Q 13. What are the encountered difficulty applying the instructions / training provided by the therapist during the COVID-19 pandemic (n = 21)**		
○ No	16	76.2
○ Yes	5	23.8

**Fig 4:**
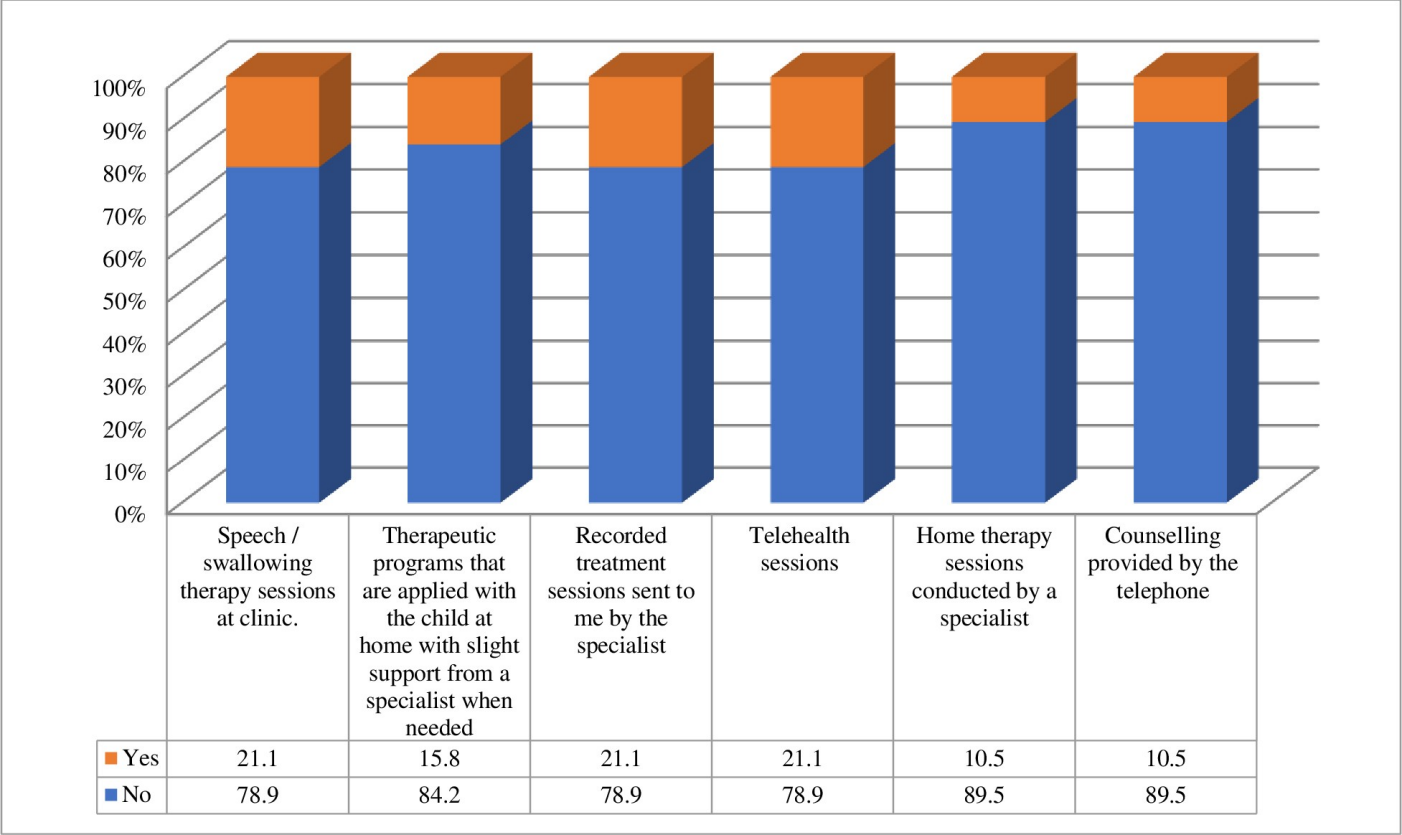
Kinds of services provided by the speech and swallowing specialist during COVID-19 (n = 21).

Those who responded “No” to (Q9) (19%, n = 21/110) were asked to choose from a list the types of services provided by the SLP during COVID-19. Among the responses, 80% reported receiving recorded treatment sessions by the SLP, 21% continued to have their sessions at the clinic, 21% received telehealth sessions, 15% applied therapeutic programs to their children themselves with a little as-needed support from their therapists, 11% had home therapy sessions conducted by the SLP, and 11% received counseling sessions via telephone (Fig 4). Seventy six percent (n = 16/21) benefited from the service the therapist provided to their child during the pandemic (Q12), while 23.8% (n = 5/21) encountered difficulties applying the instructions/training provided by the therapist during the pandemic (Q13).

### Effectiveness, applicability, and usefulness of providing speech and swallowing sessions remotely

In the final part of the survey, all respondents were asked if they wanted to have telehealth counseling sessions with SLPs (Table 5). Of the 385 respondents, 61% wanted to have this service available. Furthermore, participants who had or were suspected to have a child with SLS problems were asked to share their perspectives on the effectiveness, applicability, and usefulness of providing therapy sessions remotely. Of a total of 196 participants, 189 responded to this section.

**Table 5:** Association between having disordered child and desire of caregivers for telehealth session.

	Want to have telehealth counselling sessions		Total		P-value
	Don’t want	Want			
**Do you have any children with speech, language or swallowing disorders?**					
○ No	118(77.6)	70(30.0)	188(48.8)	84.226	< .0001*
○ Yes	28(18.4)	146(62.7)	174(45.2)		
○ May be	6(3.9)	17(7.3)	23(6.0)		

*P-value is statically significant ≤ 0.05

About 51% indicated the ineffectiveness of using the phone to provide speech therapy services, and about 34.4% of that group disagreed with the idea of providing therapy sessions via telephone rather than at clinics. Just over half of the respondents (55%) were uncertain about the usefulness of providing information over the phone, 30% chose “Agree”, and 24% chose “Disagree” (Table 6).

**Table 6:** Effectiveness, applicability, and usefulness of providing speech and swallowing sessions remotely (via phone).

	Characteristics	No	%
Effectiveness	**14. Can speech / swallowing sessions be provided through phone calls (n = 189/196)**		
	○ No	96	50.8
	○ Yes	36	19.0
	○ May be	57	30.2
Applicability	**15. In your opinion, can speech/swallowing sessions provided through phone calls replace clinical setting?**		
	○ Totally agree	7	3.7
	○ Agree	11	5.8
	○ Not sure	60	31.7
	○ Do not agree	65	34.4
	○ Totally disagree	46	24.3
Usefulne**ss**	**16. In your opinion, can the information provided over the phone can be useful?**		
	○ Totally agree	14	7.4
	○ Agree	30	15.9
	○ Not sure	104	55.0
	○ Do not agree	24	12.7
	○ Totally disagree	17	9.0

Regarding the responses to (Q18) provided in Table 7, only 38.6% of the respondents thought the video call could be used to provide therapy sessions. About 34% of the respondents agreed that video calling in therapy can be effective, while about 46% were uncertain. Finally, about half (49%) answered in the affirmative when asked, “Can speech/swallowing sessions be provided through video rather than at clinics?”

**Table 7:** Effectiveness, applicability, and usefulness of providing speech and swallowing sessions remotely (via video).

	Characteristics	No	%
Applicability	**18. In your opinion, can speech/swallowing sessions provided through video calls replace clinical setting?**		
	○ No	40	21.2
	○ Yes	73	38.6
	○ May be	76	40.2
Effectiveness	**19. In your opinion, are speech/swallowing sessions provided through video calling can be effective**		
	○ Totally agree	23	12.2
	○ Agree	42	22.2
	○ Not sure	86	45.5
	○ Do not agree	21	11.1
	○ Totally disagree	17	9.0
Usefulness	**20. In your opinion, Can the information provided over video calls be useful?**		
	○ Totally agree	35	18.4
	○ Agree	57	30.2
	○ Not sure	57	30.2
	○ Do not agree	20	10.6
	○ Totally disagree	20	10.6

## Discussion

This study has focused on caregivers’ perspectives on SLS services in Saudi Arabia, both in general and during the COVID-19 pandemic, with reference to other studies available in the literature. Also, the study investigated the willingness of caregivers to use remote services as one of the service delivery modes. Although the study has completed in Saudi Arabia, the obtained set of data examining the caregiver perspectives from the proposed questions in the survey could be meaningful to several countries and population. The reported findings revealed the importance of providing different options for health services, especially for those who do not have the opportunity to attend sessions on a regular basis, and the need to cope with this unexpected global crisis through adopting remote services across the world.

### Accessing and availability of SLS services

Over half of the respondents reported a lack of available SLS clinics in their area/city, which is consistent with an earlier study conducted by Milaat et al [2], and a third of the respondents were not aware if clinics were available. While the reason for this is unclear, it could be related to the shortage of service availability in the country and the lack of SLPs working in private clinics [33].

On the other hand, 56% of the caregivers in this study who expressed their concerns about their child’s speech had accessed SLS services. The rest of the caregivers did not access these services despite their children’s need for them. The latter results are consistent with that of [16] who reported that SLS services had not been accessed by 67.7% of the families who were apprehensive about their children’s speech. The reason for not accessing the service is not clear; however, it could be related to a lack of knowledge about the availability of services in their area, how to access the services, or even a lack of awareness of the benefits of these services, which may have impacted their access to the service [16].

Forty-seven percent of respondents reported that their children received SLS services once a week, which is less frequent than the literature’s recommended two-to-three sessions per week for children with speech sound disorders [13] and daily sessions for children with Childhood Apraxia of Speech [34]. The longest provided sessions prior to discharge were less than two months in duration. Thus, based on the frequency and length of the sessions in this study, it could be concluded that children in Saudi Arabia tend to have approximately eight sessions before discharge from SLS services. If we assume that each session lasts 30–45 minutes, then the total amount of time of the sessions before discharge would range between 4 and 6 hours; this is not enough, as indicated by Law and Conti-Ramsden [35]. Thus, it can be anticipated that the caregivers will not witness great improvements in the skills of their children’s speech and language. According to the findings of the current study 14.6% of caregivers discontinued services because they did not find any improvement in their child’s condition.

### Changes in SLS services during the COVID-19 pandemic

Assessment for, and treatment of, SLS services usually require face-to-face communication. However, due to quarantine measures and social distancing during the COVID-19 pandemic, health services, including SLS, were seriously interrupted [36, 37]. Similarly, immediate care tended to be rescheduled or managed by phone or telehealth [38]. In the current study, caregivers were asked if therapy sessions had been stopped as a response to the COVID-19 pandemic. About 80% reported that therapy sessions had been stopped and they identified different reasons, including difficulty accessing the service. This was reflected by the caregivers’ reticence to risk exposure to the virus in the health care setting, temporary suspension of therapy sessions, or closure of clinics.

It is interesting to note that, for the few respondents (n = 21) who reported continued delivery of services during the COVID-19 lockdown, 21% of children received direct sessions in the clinic and 21% received telehealth therapy, while most received recorded treatment sessions from their SLPs.

### Willingness to use remote SLS services

Based on their experiences accessing SLS services during COVID-19, different suggestions were proposed to the caregivers to evaluate their willingness to use remote SLS services. One of these suggestions was the implementation of telehealth. More than 60% of respondents were willing to use telehealth for counseling. Respondents were also asked about their willingness to use the telephone for SLS services. The major findings were as follows: over half of the caregivers opposed the use of the telephone and perceived it as ineffective, about a third did not agree with using the telephone as a replacement for clinics, and more than half were uncertain about the usefulness of providing information over the phone. These findings could be anticipated, as loss of non-verbal cues is one of the disadvantages of using the telephone in counselling [39]; however, it could be used as a temporary substitute, especially for counselling, when other options are not available.

Caregivers were generally more open to the idea of receiving video service as opposed to telephone service. This was anticipated, as the availability of non-verbal cues and visual information can support the establishment of greater rapport and trust between the caregiver and children [39, 40]. In this study, 21% of the participants would have preferred face-to-face visits with clinicians instead of video services, and 40% were uncertain. This uncertainty could be linked to a lack of previous experience about how the video service would work [41, 42]. Nearly half of the participants perceived it as effective and found the information obtained over the video calls useful.

## Conclusion

This study revealed that SLS services in Saudi Arabia are limited and that access to the service is challenging. The average number of sessions before discharge was eight in a two-month period, which is less frequent than the number of sessions recommended in the literature. Since the start of the COVID-19 pandemic, utilization of telehealth services has increased significantly. The rapidly increasing use of telehealth in the delivery of services during the COVID-19 pandemic is very promising. Moreover, caregivers in general showed a willingness to use telehealth to provide speech therapy sessions, preferring video calls over other options.

This alternative service delivery model has the potential to improve access to SLS services for children living in remote areas, to reduce the time and financial costs of traveling, and to alleviate educational and vocational burdens. The potential for using telehealth could be related to an awareness and understanding of how it can be used as an additional option for caregivers and patients. The respondent caregivers in this study had little experience using telehealth. This might be linked to the recent emergence of telehealth in Saudi Arabia, which could compromise its potential for acceptance during the current period. Thus, the findings highlight the importance of raising awareness about what the telehealth service involves so that consumers can consider their options about the services they prefer.

## Supporting information

S1 File (SAV)

S2 File (DOCX)
