# Supplementary material for: Assessment of caregivers’ perspectives regarding speech-language services in Saudi Arabia during COVID-19
Source: PLoS One. 2021 Jun 22;16(6):e0253441. doi: 10.1371/journal.pone.0253441 (PMC8219133; doi:10.1371/journal.pone.0253441)
Supplement: S2 File — (DOCX) [file pone.0253441.s002.docx]

**S2 File:**

| **Sample Size Calculation** | | | | | |  |
| --- | --- | --- | --- | --- | --- | --- |
|  | | | | | |  |
| Population size (for finite population correction factor or fpc)(*N*): | | | | | 209574 |  |
| Hypothesized % frequency of outcome factor in the population (*p*): | | | |  | 50%+/-5 |  |
| Confidence limits as % of 100(absolute +/- %) (*d*): | | | | | 5% | |
| Design effect (for cluster surveys-*DEFF*): | | | | | 1 |  |
| **Sample Size(*n*) for Various Confidence Levels** | | | | | |  |
|  | | | | | |  |
|  | **Confidence** | **Level(%)** | **Sample Size** |  |  |  |
|  | 95% |  | 384 |  |  |  |
|  | 80% |  | 165 |  |  |  |
|  | 90% |  | 271 |  |  |  |
|  | 97% |  | 470 |  |  |  |
|  | 99% |  | 662 |  |  |  |
|  | 99.9% |  | 1078 |  |  |  |
|  | 99.99% |  | 1504 |  |  |  |
|  | | | | | |  |
| Equation | | | | | |  |
| Sample size ***n* = [DEFF*Np(1-p)]/ [(d^2^/Z^2^_1-α/2_*(N-1)+p*(1-p)]** | | | | | |  |

Results from OpenEpi, Version 3, open source calculator--SSPropor
Print from the browser with ctrl-P
or select text to copy and paste to other programs

# Reference for population size: The National and Regional Prevalence Rates of Disability, Type, of Disability and Severity in Saudi Arabia-Analysis of 2016 Demographic Survey Data. [*Bindawas SM*](https://www.ncbi.nlm.nih.gov/pubmed/?term=Bindawas%20SM%5BAuthor%5D&cauthor=true&cauthor_uid=29495546) *et al.* [Int J Environ Res Public Health.](https://www.ncbi.nlm.nih.gov/pubmed/clipboard) 2018 Feb 28;15(3). pii: E419. doi: 10.3390/ijerph15030419.
